# Enhancing the learning of evolutionary anthropology skills by combining student‐active teaching with actual and virtual immersion of Master's students in fieldwork, laboratory practice, and dissemination

**DOI:** 10.1002/ece3.8825

**Published:** 2022-04-15

**Authors:** Priscilla Bayle, Dominique Armand, Maryelle Bessou, David Cochard, Christine Couture, Marie‐France Deguilloux, Catherine Ferrier, Cathy Haget, Jacques Jaubert, Christopher Knüsel, Stéphanie Martins, Éric Pubert, Stéphane Rottier, Antoine Souron, Cédric Beauval, Arnaud Caillo, Bruno Dutailly, Thomas Girault, Malo Hesry, François Lacrampe‐Cuyaubère, Ronan Ledevin, Caroline Masset, Miriam Mesa‐Saborido, Pascal Mora, Xavier Muth, Raphaël Pinson, Adrien Thibeault, Marc Thomas, Nicolas Vanderesse, Jean‐Guillaume Bordes

**Affiliations:** ^1^ 27086 UMR5199 CNRS, MC, PACEA University of Bordeaux Pessac France; ^2^ 27086 Mission d'Appui à la Pédagogie et à l'Innovation University of Bordeaux Bordeaux France; ^3^ Archéosphère SARL Quillan France; ^4^ 27075 UMS 3657 CNRS, Archéovision Bordeaux Montaigne University Pessac France; ^5^ Musée National de Préhistoire (MNP) Les Eyzies France; ^6^ Get In Situ SARL Riex Switzerland; ^7^ UMR 5608 CNRS, TRACES University of Toulouse Jean Jaurès Toulouse France

**Keywords:** 3D imaging, evolutionary anthropology, fieldwork, laboratory practice, student engagement, student‐active teaching

## Abstract

Higher education in evolutionary anthropology involves providing students with in‐depth knowledge of biological and cultural heritage sites and collections that are frequently inaccessible. Indeed, most sites, fossils, and archaeological remains can be visited or manipulated only rarely and solely by specialists with extensive experience. Owing to the development of 3D and medical imaging techniques, this fragile heritage is now more widely accessible, and in a dynamic way. However, exclusive adoption of virtual teaching and learning has a negative impact on student engagement and, naturally, on exchanges with instructors, and thus cannot be used without some reservations. In the ITAP (*Immersion dans les Terrains de l’Anthropologie biologique et de la Préhistoire*) project of the higher education STEP (*Soutien à la Transformation et à l’Expérimentation Pédagogiques*) transformation program at the University of Bordeaux, we combine student‐active teaching with Master's students fully immersed in ongoing fieldwork, laboratory study, and dissemination of research results in order to develop more individually shaped learning curricula and to foster both professional and new interdisciplinary skills. Here, we present examples of experiments conducted in the ITAP project using both authentic and virtual collections of archaeological, experimental, and reference materials that help to break down the barriers between research activities and higher education, as well as providing a more general appraisal of the appropriate use of virtual tools in higher education by combining them with real‐life situations.

## Graphical abstract

The article describes examples of novel teaching methods that we have developed in our Master's program in evolutionary anthropology, where we combine student‐active teaching with Master's students fully immersed in ongoing fieldwork, laboratory study, and dissemination of research results.

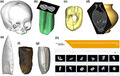

## 1 INTRODUCTION: EMBRACING OR FACING THE DIGITAL REVOLUTION IN HIGHER EDUCATION

In a world where digital technology occupies a prominent place, its use and the increased variety of pedagogical practices it entails have gradually been integrated into university programs. As a tool with considerable cognitive benefits, the impact of digital technology on student learning and success needs to be taken into consideration and regularly assessed as technologies rapidly evolve: for example, their impact on student attention span, the potential disconnection from reality, the lack of exchange with instructors, and the limits of virtual exchanges, but also their role in developing a taste for learning, the acquisition of 21^st^‐century skills, access to new data, and intellectual stimulation (Karsenti, 2018). Researchers have been working on all these issues for several decades already, but they have clearly been amplified as a result of the global health crisis in 2020 (Point, 2020).

Natural and social sciences are based on observation and the quantification of physical or “concrete” data. Situated at the crossroads between observation and quantification, evolutionary anthropology is no exception. Delivering courses in both biological anthropology and prehistoric archaeology, the University of Bordeaux fully embraces the tradition of holistic approaches to examining the human past, training for future higher education employment and research, field archaeology and bioarchaeology, heritage curation, medico‐legal studies, and broad public dissemination professionals in its dedicated Master's program. In order to give students the opportunity to learn the required professional skills, it is essential to expose them to real‐life situations during field trips and excavations and to permit them to manipulate research materials and objects. Furthermore, teaching in ways that include fieldwork, practical activities, and experiments enhances student learning outcomes, stimulating senses linked to several areas of the brain (Gya & Bjune, 2021; Nabors et al., 2009; Willis, 2007). Such activities are mainly conducted in groups. This promotes the development of problem‐solving and thinking skills and typically involves a communal atmosphere, building student confidence and potentially reducing the release of stress hormones in their brains, which in turn removes a factor that can impede learning (Owens & Tanner, 2017). In addition, conducting real fieldwork and experiments enhances student engagement and the emergence of skills by offering them the possibility to participate as scientists and to conduct joint projects with instructors (Hole, 2017). Moreover, evolutionary anthropology is relatively unique in that it relies on heritage sites and materials (e.g., cave paintings, hominin fossils, unique archaeological artifacts), as well as on extant data (notably human biological data, rare animal taxa, unique reference collections) that are scattered across the globe and can only rarely be accessed or manipulated. While various reproduction techniques have existed for some time (e.g., reconstructions, casts...), in many cases they cannot be used for materials that are too fragile or too complex. Above all, there is often significant degradation of the original specimens involved in their production. Furthermore, the cost of conducting practical activities and off‐campus fieldwork courses is comparatively high. As a result, student training is not only impeded by the preservation of rare and fragile materials and ethical concerns (e.g., personal data protection, confidentiality, taboos regarding human bodies) but also cuts to university budgets. Together with the impact of the current pandemic, this makes it increasingly challenging to rely only on practical in‐person learning activities to train future evolutionary anthropology professionals.

Consequently, virtual imaging has been a game‐changer in the discipline as it has opened up broader access to the scarce heritage composed of a few sites and fragile fossils and artifacts, whose conservation and study mirror major societal issues such as climate change, reduced biodiversity, and resource scarcity (Macchiarelli & Weniger, 2011; Semal et al., 2005). In addition to this, it has permitted broader access to otherwise inaccessible specimens and sites which currently provide the raw material for many research projects. Without replacing direct access to sites and materials when possible, their virtual observation or manipulation can efficiently complement such access. Indeed, in addition to their essential role in immortalizing heritage sites and collections, non‐invasive imaging techniques permit full access to a variety of sites, fossils, and visualization of external and internal constituents of objects and biological structures from around the world. For example, they can reproduce extremely faithful images of surfaces thanks to meshed and/or textured 3D models at different scales, from a macro/meso‐scopic scale (e.g., object or ground‐based photogrammetry; reviewed in Magnani et al., 2020) to a micro/nano‐scopic one (e.g., confocal microscopy; reviewed in Calandra et al., 2019; Schmidt et al., 2020), and can make their internal structures accessible, and at a very high resolution (e.g., X‐ray, synchrotron radiation, or neutron microtomography; reviewed in Bayle et al., 2011; Macchiarelli et al., 2013; Tafforeau et al., 2006; Zanolli et al., 2020).

While these digital advances offer fantastic possibilities in evolutionary anthropology, their potential negative effects in higher education on anchoring students to real‐life situations, the potential lack of communication with instructors, as well as the very high carbon footprint for the production and storage of increasingly heavy multimedia data, cannot be ignored. Archiving several copies of heavy 3D raw data files and models on different servers for years has a substantial carbon footprint, which easily dwarfs the footprint associated with transporting students to fieldwork locations within the region but not immediately adjacent to university urban settings. In order to make the best use of the potential of digital technology for student learning, without denying its negative effects, we therefore decided to systematically connect it to real‐life manipulations and situations in the ITAP (*Immersion dans les Terrains de l’Anthropologie biologique et de la Préhistoire*) project under the auspices of the higher education STEP (*Soutien à la Transformation et à l’Expérimentation Pédagogiques*) transformation program at the University of Bordeaux.

Here, we present examples of experiments conducted in the ITAP project using both actual and virtual collections of archaeological, experimental and reference materials, as well as full student immersion in fieldwork, laboratory and broad public dissemination settings that help break down the barriers between higher education and the professional world. At a time when practical, in‐person learning activities are under threat in universities worldwide, we contribute to a general appraisal of the appropriate use of virtual tools in higher education by combining them with real‐life research situations.

## 2 PROVIDING THE TIME NECESSARY FOR FIELD AND LABORATORY WORK

**FIGURE 1:**
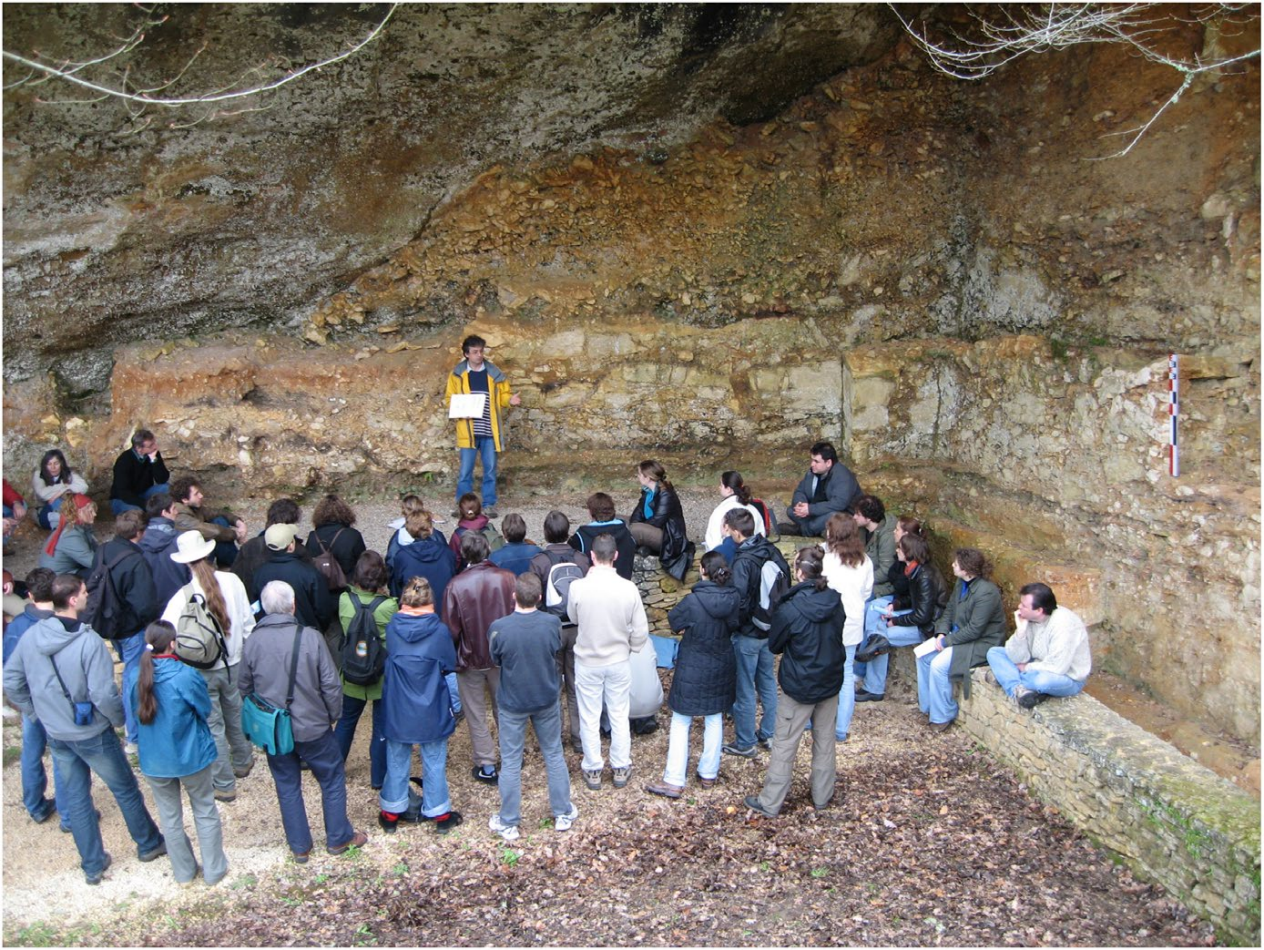
Guided excursion to the famous prehistoric site of La Ferrassie (Savignac‐de‐Miremont, Dordogne, France) which yielded a long Paleolithic stratigraphic record and several Neandertal burials

Located in a region renowned for its well‐preserved and numerous prehistoric archaeological World Heritage‐listed UNESCO sites, the Master's program in biological anthropology and prehistoric archaeology at the University of Bordeaux has a long tradition of immersive teaching, offering field schools on sites that have been studied for over a century to gain insights into biological and cultural evolutionary history. Our institutional network, which includes the Ministry of Culture, the Musée National de Préhistoire (MNP, Les Eyzies), the Institut national de recherches archéologiques préventives (Inrap), other public and private salvage archaeology organizations and the Pôle d'interprétation de la Préhistoire (PIP, Les Eyzies), provides a unique opportunity for students to engage with a diversified professional and socioeconomic environment, especially during field trips (Figure 1).

The students complete a three‐week fieldwork course in the first year of a two‐year Master's program. This off‐campus course is based on the excavation of archaeological sites. The aim is to train students in the ins and outs of fieldwork through a hands‐on approach which involves project design (including legislative and regulatory constraints), excavation set‐up, excavation methods and tools, data collection and analyses, and the design and writing of a final excavation report. Depending on their specializations, students can choose between two fieldwork courses. One field school takes students to the excavation and associated fieldwork of a prehistoric site. Here, the research objectives are to reconstruct the biological and cultural evolution of past hominins based on the study of their biological identity and lifeways. For the last five years, this field school has been conducted at the Pleistocene hunters‐gatherer base camp of Le Piage (Fajoles, Lot, France) (Bordes et al., 2008). In this paper, we illustrate our experiential off‐campus learning practices with examples from this fieldwork course.

**FIGURE 2:**
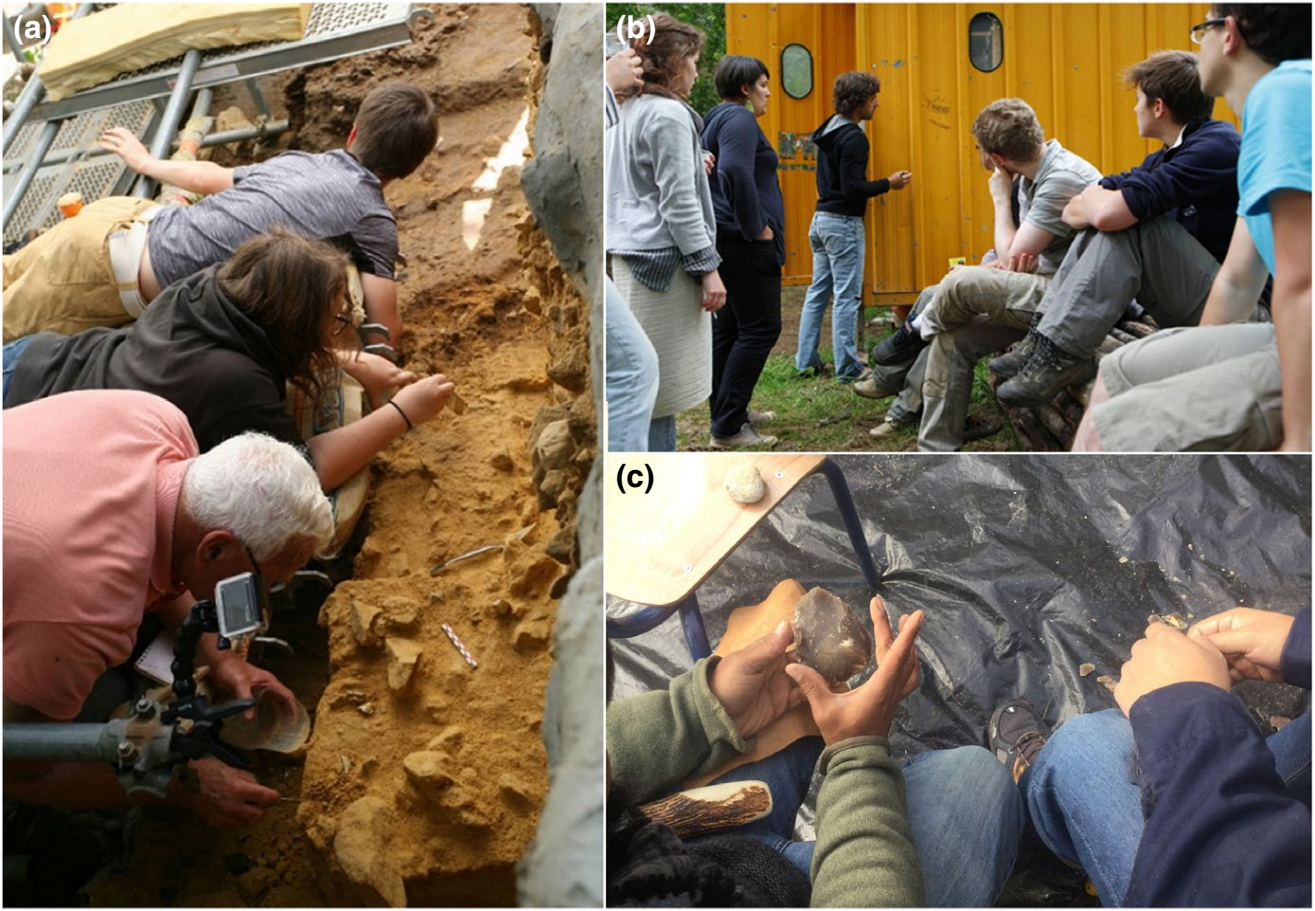
(a) Excavation of Aurignacian levels by Master's students at Le Piage; (b) Improvised exchange on the solifluction process which mixed the Solutrean and Badegoulian levels at Le Piage; (c) Exchange during a flint‐knapping actualistic experiment

Le Piage is located at the foot of a Coniacian limestone cliff containing numerous karstic cavities. Excavated between 1958 and 1968, and again since 2004, the deposits have yielded Mousterian, Châtelperronian, Protoaurignacian, Early and Late Aurignacian, Solutrean, and Badegoulian cultural complexes occurring from 55,000 to 20,000 years BP (Bordes et al., 2008; Champagne & Espitalié, 1981). This multi‐stratigraphic site provides archaeological strata that have revealed very rich lithic and faunal remains, as well as adornments and human remains. Appearing around 42,000 years ago in Southern Europe, the Protoaurignacian cultural complex is extremely important to the debate on the settlement processes of modern humans in Western Eurasia as it corresponds to a period when Neandertals still occupied this region (Hublin, 2015). Few Protoaurignacian levels have been found up to the present time. Le Piage is among the few sites that have yielded Protoaurignacian levels and is one of the extremely rare sites to have yielded human remains associated with this archaeological cultural complex (Benazzi et al., 2015). These characteristics make it a key piece of the puzzle concerning the makers of tools and artifacts during a period marked by dramatic biological and cultural changes in Eurasia (Hublin et al., 2020; Zilhão et al., 2015). Furthermore, complex post‐depositional natural processes occurred at the site, notably solifluction which mixed the Solutrean and Badegoulian cultural material. All these factors make this site of equal and major interest for geoarchaeologists, archaeologists, paleontologists and biological anthropologists. Every year, students are divided into groups, with each group responsible for excavating a specific area of the site (generally a quarter of a square meter) and handling the associated post‐excavation processing, as well as writing the excavation report for that part of the site (Figure 2a). Students are invited to propose excavation strategies for the area they are entrusted with and to discuss their ideas with the supervisory team. This experiential off‐campus learning fosters exchange with instructors on observations made directly by the students in the field, giving them a better grasp of the related questions (Figure 2b). By giving students the opportunity to excavate renowned archaeological sites and to work with authentic archaeological material, the fieldwork course offers them a unique opportunity to make new discoveries, sometimes of major importance to our knowledge of human evolution. Giving them enough time to manage the entire process, to actively work as a team, and to optimally interact with all the specialists involved in the fieldwork (Figure 2c) increases their engagement to reach common objectives, placing this course at the most student‐active end of the off‐campus experiential learning spectrum (for a recent review, see Gya & Bjune, 2021). The positive effects of this practice have been highlighted in recent years by the students’ ability to generate new questions during fieldwork, reveal new findings, and contribute to excavation reports and further dissemination of results (Bordes et al., 2018, 2019, 2020; Faivre et al., 2015; Rottier et al., 2017).

Furthermore, during the off‐campus course at Le Piage, students also take part in several post‐excavation activities in which the teaching material is authentic archaeological material from Le Piage or from other nearby prehistoric sites. For instance, students took part in research related to Sirogne cave (Rocamadour, Lot, France), where lithic artifacts and fossil remains, including about 90 Neandertal remains belonging to at least 10 individuals, were found during the excavations conducted since 2013 in Middle and Late Pleistocene deposits of the site (Bayle, Albouy, et al., 2017; Bayle et al., 2014). Students carried out a study on the effect of occlusal wear on the identification of single‐rooted teeth of both humans and bears. Indeed, as several diagnostic features are located on the tooth crown (shape, enamel thickness), it can be difficult to distinguish bear incisors (especially the first and second lower incisors) from human incisors and canines when occlusal surfaces are worn. Examples of confusion have been found from iconic prehistoric sites (e.g., Arcy‐sur‐Cure; Leroi‐Gourhan, 1958) and from recent discoveries of teeth identified as human (e.g., at Axlor; Gómez‐Olivencia et al., 2019). At Sirogne, this issue is of major importance as the faunal spectrum is heavily dominated by bears, mostly represented by isolated dental remains, and more than 30 of the Neandertal remains discovered to date are isolated deciduous or permanent incisors or canines. For this preliminary research work related to the current fieldwork at Sirogne, students were entrusted with original, freshly excavated authentic human and faunal remains. They were very enthusiastic and contributed original results to the quantitative distinction between worn human and bear teeth. Their work was the focus of a contribution co‐authored by them in the Sirogne excavation report (Bayle, Maureille, et al., 2017).

Given the uniqueness of high‐profile cultural heritage resources, however, access to authentic sites and materials is rare. To be suitably prepared, students have to learn practical skills which cannot be acquired solely through face‐to‐face teaching due to the limited time available. Under the guidance of instructors, students are therefore entrusted with the preparation of educational resources to be taken home. For example, toolkits were created for major categories of stone *débitage* found in Paleolithic sites with knapping products experimentally replicated and/or coming from collections of unknown archaeological origin. The students can thus practice identifying stone artifacts at home, as well as conjoining exercises between parts from a same block of raw material, allowing them to reconstruct the *chaîne opératoire* employed by prehistoric humans (see the importance of such practices in Audouze & Karlin, 2017). In addition to the use of physical materials to be taken home, we rely on the considerable potential of 3D virtual imaging to increase the prerequisite skills of students required to work with original sites and specimens.

## 3 PREPARING FIELD AND LABORATORY WORK USING VIRTUAL IMAGING

Owing to their non‐invasive nature and high potential for real‐time exploration and quantification of structures, 3D imaging techniques are now widely used in evolutionary anthropology. A striking illustration can be found in the Lascaux Paleolithic decorated cave, access to which is extremely limited for conservation reasons, but which can now be visited in 3D (see https://archeologie.culture.fr/lascaux/fr/visiter‐grotte‐lascaux).

**FIGURE 3:**
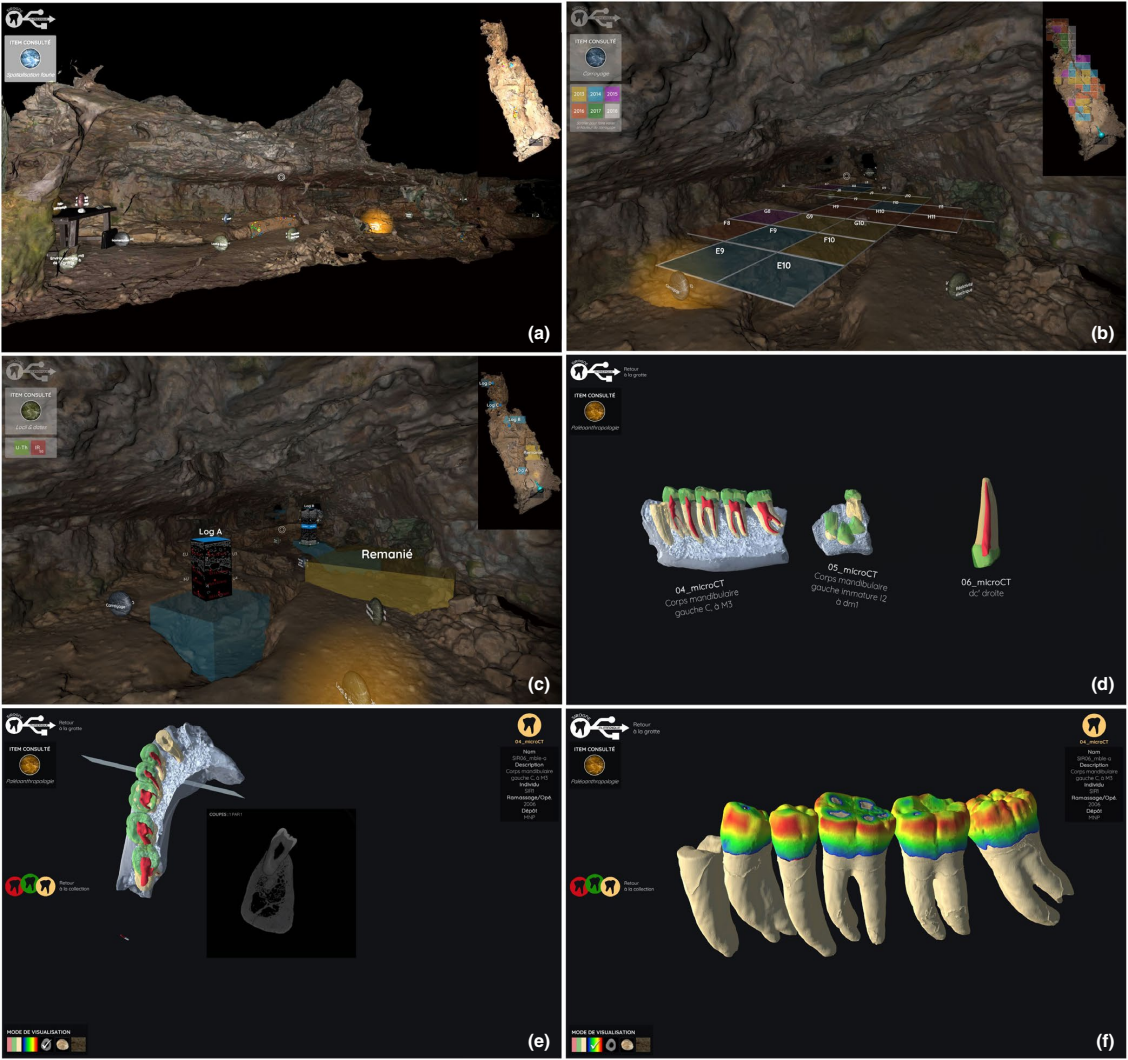
Screenshots of the “Virtual Sirogne” application: (a) the 3D model of the cave and of the different activities with a “wall pass effect” passing through the east wall (zenithal section view of the cave in the top right‐hand corner); (b) the grid with the first year of excavation of the different squares located on the 3D model of the cave as viewed from its entrance; (c) the stratigraphic layers located on the 3D model of the cave seen from its entrance; (d) the home page of the human fossil module showing microtomographic‐based models; (e) the exploration through the microtomographic‐based model of a fossilized human left mandibular dental arcade with extraction of a virtual slice; (f) the teeth virtually extracted from the microtomographic‐based model of a fossilized human left mandibular dental arcade and showing the variation of enamel thickness ranging from “thin” blue to “thick” red (development of the application by Archéosphere)

In addition to its value in the virtual exploration of sites that are rarely or not at all accessible, 3D imaging can be used to integrate archival data in archaeological fieldwork (Discamps et al., 2016) or to allow students or volunteers to explore a site before they participate in its actual excavation. With this in mind, in 2018, we began to develop the “Virtual Sirogne” application which provides a dynamic introduction to this prehistoric site (Figure 3). The application includes photogrammetric models of the whole cave, its fill, objects, non‐human animal fossils, as well as microtomographic‐based images and 3D models of the Neandertal remains. This practical experience not only gives access to fossilized human remains and to their external and internal structures in a way that is unique in higher education, in other words, through full access to the remains virtually presented within the 3D model of the site of their discovery, but also offers prior immersion in a real‐life archaeological excavation. Thus, students and volunteers participate in the fieldwork only after acquiring in‐depth knowledge of the context and challenges of the field research being conducted. Moreover, the scientific team specialists can discuss the progress of their research better, even remotely from the site, providing opportunities for more frequent updates and brainstorming. The application is currently only available to students in our Master's program who provide regular feedback and thus contribute to its development. With some adaptation, it will also be a valuable tool for disseminating our findings to the public. A video showing the cave, its environment, some of the 3D models, and educational resources is available at: https://youtu.be/BYhtfV_5R44.

**FIGURE 4:**
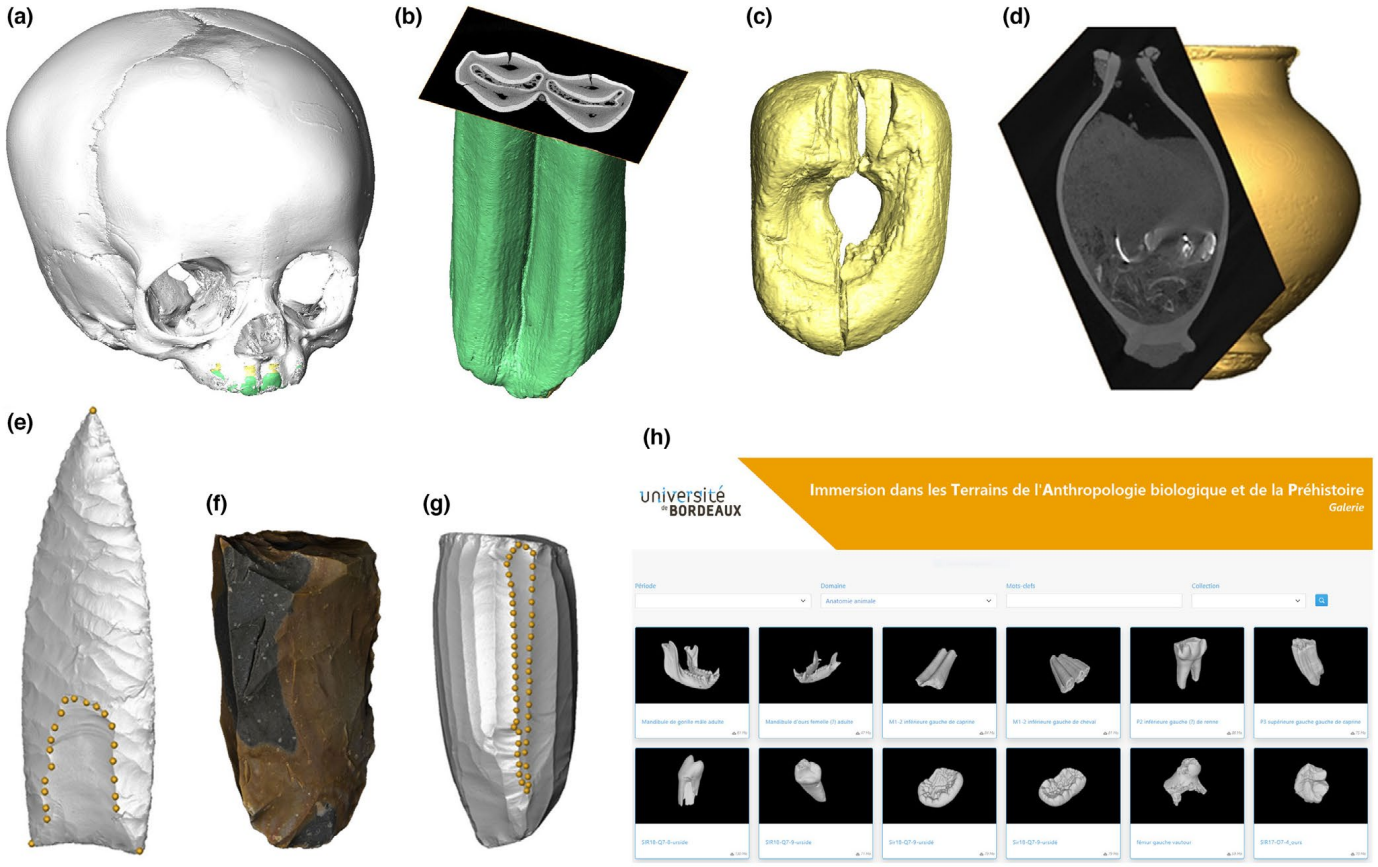
ITAP (*Immersion dans les Terrains de l’Anthropologie biologique et de la Préhistoire*) 3D gallery on the educational platform of the University of Bordeaux (h), and examples of data included in it (images not to scale): (a) microtomographic‐based 3D model of a human neonate cranium from our reference collection, with dental tissues in green and yellow, and bone in grey; (b) microtomographic‐based 3D model of a caprin lower left first or second molar from the Paleolithic site of Sirogne (Lot, France), and virtual slice showing the internal dental structure; (c) microtomographic‐based 3D model of a 4 mm‐long hard osseous material bead from the Paleolithic site of Le Piage (Lot, France); (d) microtomographic‐based 3D model of a funerary urn from the Iron Age necropolis (burial ground) of Pouyet Sud (Landes, France), and virtual slice showing its contents (soil, bones and metal); (e) microtomographic‐based 3D model of an experimental replica of an obsidian Paleoindian point, with landmark positions indicated; (f) photogrammetric‐based 3D model of an experimental replica of a flint core; (g) microtomographic‐based 3D model of an experimental replica of an obsidian core, with landmark positions indicated

In addition to those displayed in the “Virtual Sirogne” application, the fossilized and modern teeth and bones, as well as archaeological and reference lithic and bone artifacts, and personal ornaments studied by laboratory members have now been digitized in 2D and 3D at different resolutions as part of the ITAP project. Indeed, fossils and artifacts are increasingly detailed with high‐resolution 3D imaging techniques, and their applications in evolutionary anthropology have multiplied and become more varied over the last two decades (Cunningham et al., 2014). In particular, photogrammetry and confocal microscopy have been widely used to quantify alterations preserved at micro‐ and nano‐scopic scales on the surface of materials. The applications of these techniques permit, for instance, (1) study of cut marks and taphonomic alterations on bone surfaces, (2) identification of the functional history of stone and bone tools and personal ornaments through use‐wear analysis, (3) reconstruction of the diet and environment of past human and non‐human animal groups through dental macro‐ and microwear analysis, and (4) elucidation of biological processes, such as growth and its disturbances through the study of growth increments in teeth and bones (Calandra et al., 2019; Galland et al., 2018; Li et al., 2020; Magnani et al., 2020; Maté‐González et al., 2017; McGrath et al., 2021; Rosso et al., 2017; Schmidt et al., 2020; Stemp, 2014; Yravedra Sainz de los Terreros et al., 2019). Furthermore, other 3D techniques such as X‐ray, synchrotron radiation, and neutron microtomography have been widely used to non‐invasively study the internal structure of bones and teeth, which holds a significant amount of valuable information for providing taxonomic identification, reconstructing phylogenetic relationships, and assessing adaptive strategies in past groups (among others, see studies by Bayle et al., 2010; Beaudet et al., 2016; Benazzi et al., 2011; Crevecoeur et al., 2014; Détroit et al., 2019; Jaeger et al., 2011; Kivell, 2016; Le Luyer et al., 2016; Macchiarelli et al., 2004; Olejniczak et al., 2008; Rossi et al., 2004; Skinner et al., 2008; Stoessel et al., 2016; Tafforeau & Smith, 2008; Zanolli et al., 2019). It has therefore become essential to enable students in evolutionary anthropology to acquire the skills of 3D image analysis. To this end, we have adopted face‐to‐face teaching in the image processing laboratory, where students practice under guidance and conduct investigations using 3D models of fossils and artifacts. However, due to time constraints, students are encouraged to individually access a dedicated space on the educational platform of the University of Bordeaux, where they can explore the 3D collection of digitized teeth, bones, and artifacts, together with a large collection of photographs and radiographic images (Figure 4). Since the early 2010s and following the pioneering initiatives of 3D data‐sharing on digital media (Seidler et al., 1999), online repositories have multiplied, and there are now many platforms that share 3D data for evolutionary anthropology research and/or didactic purposes (see Lebrun & Orliac, 2017). One of the best‐known platforms is MorphoSource (Boyer et al., 2017) but, among others, we can also cite NESPOS (https://archiv.neanderthal.de/data/; Macchiarelli & Weniger, 2011; Semal et al., 2005), the Digital Morphology Museum (http://dmm.pri.kyoto‐u.ac.jp/dmm/WebGallery/index.html), the Human Fossil Record (https://human‐fossil‐record.org/), the Laetoli Production Vertébrés application (https://laetoli‐production.fr/fr/works/12), the ESRF (European Synchrotron Radiation Facility) heritage database for paleontology, evolutionary biology, and archaeology (http://paleo.esrf.eu/), the 3D Cabinet de Curiosités of the Muséum national d’Histoire naturelle (https://cabinetdecuriosites3d.mnhn.fr/), Aves 3D (https://www.aves3d.org/), Virt. Os (https://virtos.archeogrid.fr/; Coqueugniot et al., 2020), and the MorphoMuseum (http://morphomuseum.com/; Lebrun & Orliac, 2017), the latter being dedicated to both storage and publication. Moreover, several online platforms include 3D models among other data (e.g., AfricanFossils.org at https://africanfossils.org/and the Collections de la Technothèque at https://teknotek.pretech.cnrs.fr/s/fr/page/accueil). Students are provided with the links to these platforms and are encouraged to visit them. They also receive information on the potential impact of X‐ray and synchrotron radiation on ancient DNA and ESR dating results, as well as guidelines for avoiding or limiting them (Duval & Martín‐Francés, 2017; Immel et al., 2016). The archives on the educational platform of the University of Bordeaux include 3D models specific to our field and laboratory research, intended for use as teaching resources associated with online exercises. The platform, which is still under development, will be soon available with the other online pedagogical resources of the University of Bordeaux. Furthermore, a video is included to show all the steps of a microtomographic analysis, from the loan of the specimen to be scanned from the MNP (in this example, a Neandertal mandible from Sirogne cave) to image acquisition with the X‐ray microtomographic equipment available in the laboratory, which has restricted access and is therefore not normally accessible to groups of Master's students. The video highlights both conservation and scientific aspects of the research and is available online with English subtitles at: https://www.canal‐u.tv/video/universite_de_bordeaux/analyses_3d_par_microscanner_pour_l_anthropologie_biologique_et_l_archeologie.57745.

Besides “Virtual Sirogne”, other applications using virtual models are also available to students in preliminary versions (virtual comparative anatomy and virtual microscopy), while others are still in progress (lithic object manufacturing and excavation of a human burial), or are planned for the near future (litho‐ and archaeostratigraphy of a Paleolithic site and visit to a decorated (with parietal art) Paleolithic cave). For instance, the first plug‐in of the virtual microscope permits observation and quantification of dental microstructures that are hidden within the tooth and are thus not directly accessible, despite being of the utmost interest in evolutionary anthropology. Indeed, they provide a faithful record of growth and its disturbances in past human and non‐human animal groups, contributing unique information in forensic and bioarchaeological contexts: pre‐ and post‐natal conditions, interactions between individuals (maternity), interactions between individuals/environments (nutrition, stress events, hunting strategies), age‐at‐death, etc. (Dean, 2010). However, since access is limited owing to space constraints in the laboratory, learning the laboratory skills necessary to extract such data was only possible late in student study programs, typically not before Master's or PhD thesis research. With this application, students can now learn the basics of dental histology applied in evolutionary anthropology in a dynamic way earlier on in their program of study. By watching a video made in the mineralized dental tissues technical area of the laboratory, they can also learn about the main steps involved in preparing dental hard‐tissue thin slides before they are observed under the microscope. The video is available online with English subtitles at: https://www.canal‐u.tv/video/universite_de_bordeaux/realisations_de_lames_minces_pour_l_etude_des_microstructures_dentaires.57741. Other videos are currently being edited or are in preparation to give students virtual access to laboratory instruments, including confocal microscopy and sample preparation equipment for paleogenetic analysis, which by their very nature have restricted access, early on in their study programs.

## 4 RECONCILING VIRTUAL AND REAL‐TIME LEARNING METHODS VIA 3D PRINTING FOR FIELDWORK SETTINGS

The virtual and real‐time learning can be fully reconciled to make the most of their complementary advantages. One example would be to use 3D printing as an educational resource during fieldwork. Guided by the sensitive nature of the research currently conducted at Le Piage, we decided to produce high‐resolution 3D replicas of specimens that can be used in a hands‐on manner in fieldwork settings (called the “*mallette tout*‐*terrain*” or “all‐terrain study case”).

Indeed, current fieldwork at Le Piage has reached the Protoaurignacian level (Bordes et al., 2019), significantly increasing the probability of finding other human remains in the area where three skeletal remains of a human neonate were previously recovered (Beckouche & Poplin, 1981; Maureille et al., 2007). Together with two isolated deciduous incisors found in northern Italian sites, the remains are the only human remains associated with the Protoaurignacian available for study. Given the scarcity of human remains attributed to the period of the arrival of modern humans in western Europe and the demise of the Neandertals, and their highly debated association with archaeological cultural material in most sites (Bar Yosef & Bordes, 2010; Benazzi et al., 2011, 2015; Gravina et al., 2018; Higham et al., 2011; Zilhão et al., 2015), the neonate human remains found at Le Piage are of utmost importance.

**FIGURE 5:**
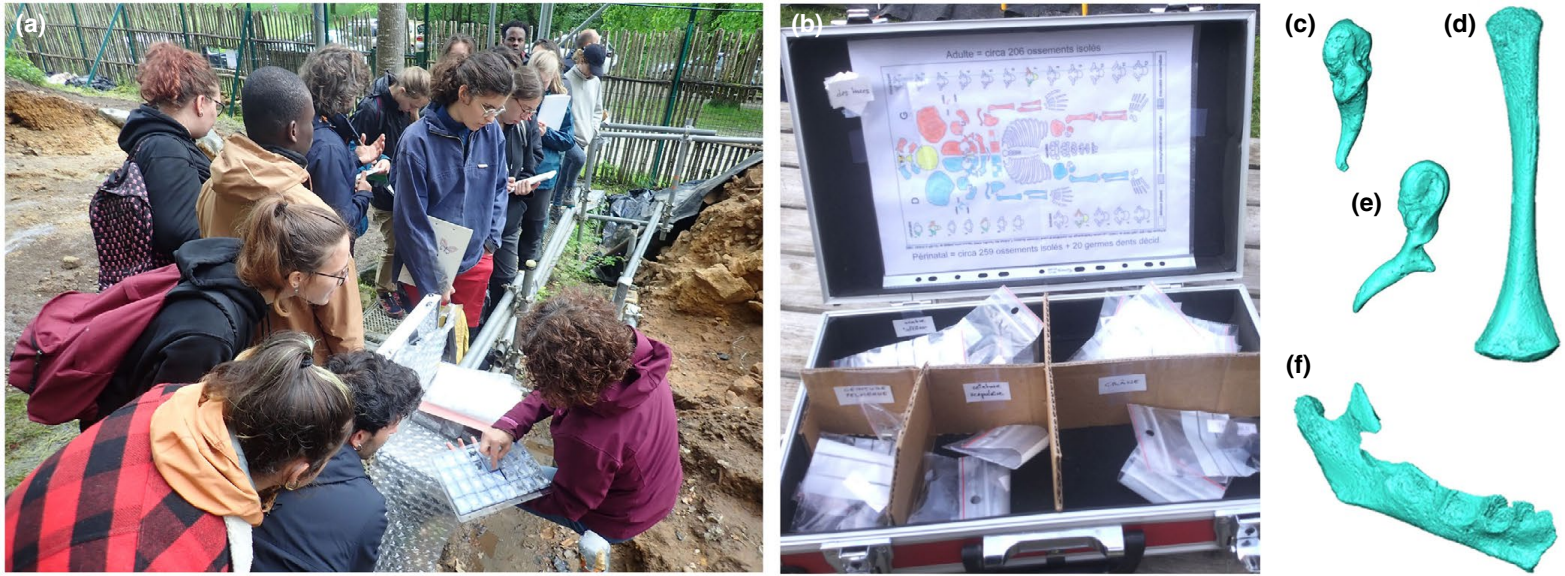
Use of high‐resolution 3D replicas in fieldwork settings: (a) presentation of the microtomographic‐based 3D replicas after opening the site on the first excavation day; (b) educational toolkit containing 3D replicas of a human neonate skeleton from our reference collection; and examples of 3D virtual models before printing of (c) the right malleus, (d) the left humerus, (e) the right incus, and (f) the right hemimandible of the human neonate

The importance of their association with the stratigraphic and archaeological context means that being able to recognize such remains in situ during excavations rather than during the post‐excavation sieving process of the sediment is fundamental to accurately associating human remains with their chronocultural context. As juvenile osteology is very complex, particularly for neonates (e.g., Scheuer & Black, 2004), developing the ability to recognize such human remains requires years of practice, which is impossible given the time constraints in undergraduate and graduate courses. In this context, Master's students, under the guidance of the fieldwork coordinators and paleoanthropologists in the scientific team, were entrusted with assembling an educational toolkit made of high‐resolution 3D replicas to be taken into the field. To this end, they were entrusted with microtomographic‐based replicas of a complete modern human neonate skeleton and the three fossil neonate remains (left *pars lateralis* of the occipital, right tibia, and left ulna) found during excavation by Champagne and Espitalié (1981) (Figure 5). The effect of the experiment was extremely positive as demonstrated by the success of the excavation. While no human remains were found, the students were highly motivated and fully embraced their responsibility in preparing the educational toolkit and making the best use of it. In particular, they were able to distinguish cervid juvenile remains from those of humans.

## 5 THE YEARLY MASTER’S STUDENTS SYMPOSIUM

**FIGURE 6:**
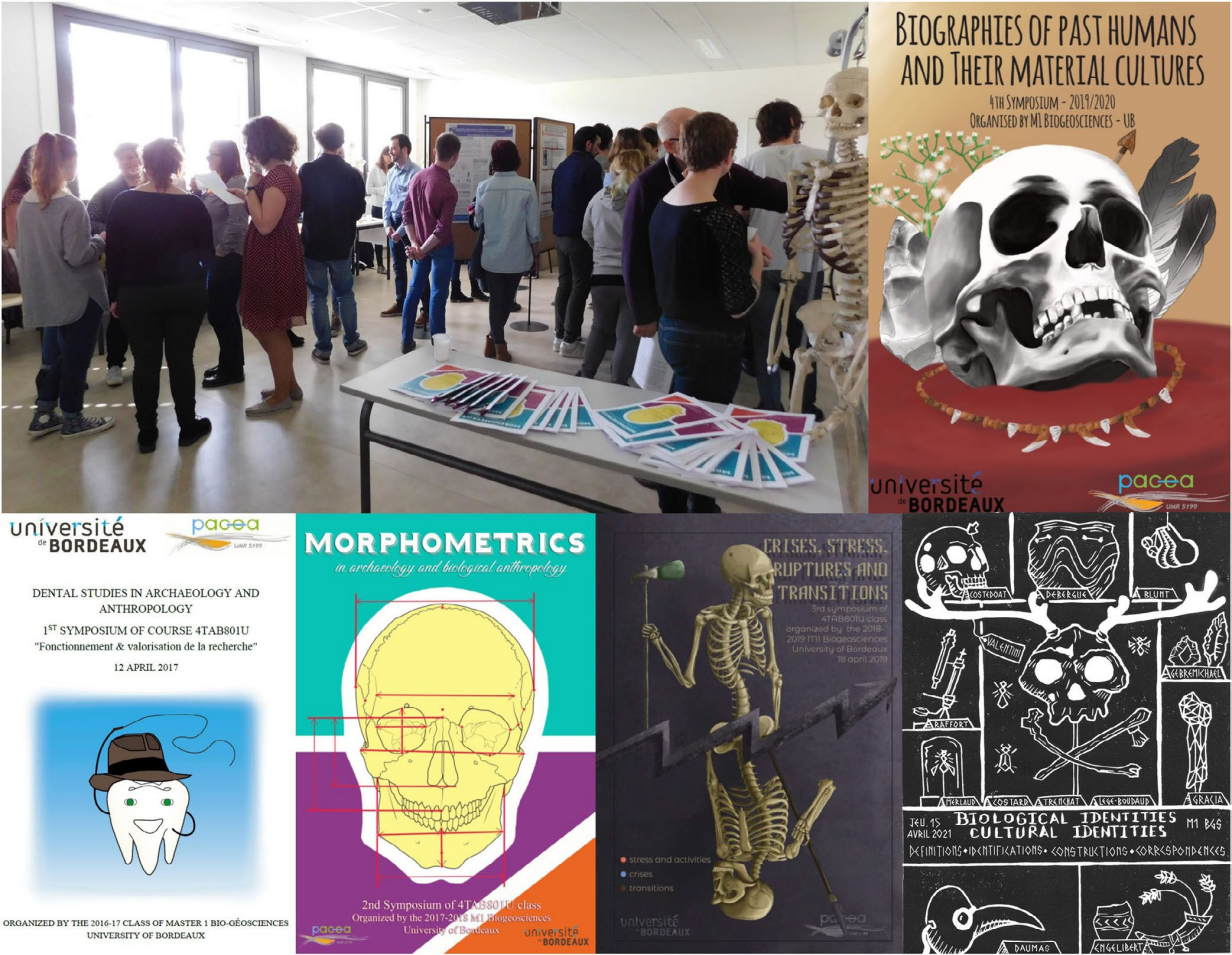
Second Master's student symposium in evolutionary anthropology (in 2018), and cover pages of the first, second, third, fourth, and fifth books of abstracts

In parallel with their virtual and on‐site immersion in both the field and laboratory, Master's students complete another student‐active experiment which takes them to the final phases of the scientific process, in other words, disseminating results. To this end, following an introduction on how current scientific research works (designing a project, building a team, seeking funding, dissemination, the workings of the peer‐review process), they organize a poster symposium based on a major research topic in evolutionary anthropology. Students partake in both the symposium organizational and scientific committees, assuming all the roles involved in organizing a symposium and, more generally, an event, including an editorial committee, communications, internal organization, purchasing and finances, graphic design, and organization of the day itself. They must actively work together and interact with departmental members during the half‐day poster symposium held in the PACEA teaching laboratory. The entire course and the symposium are conducted in English to bring the students as close as possible to a real‐life situation. Before the symposium, each student interacts with a scientific referee in the laboratory to prepare an abstract based on selected relevant references, and a poster on the subject of their choice from a list of topics provided each year. They also experience the peer‐review system by evaluating each other's abstracts. The students are all highly motivated, as seen in the books of abstracts produced in previous years that follow professional standards, and by the success of the symposia, which reflects the atmosphere of the scientific exchanges between students and researchers (Figure 6). Moreover, the outcomes of this exercise are also extremely positive as the students frequently continue to work on their chosen topic in the context of a Master's thesis, often under the supervision of their symposium referee. All of the contributions for the 2021 symposium, which was exceptionally conducted online due to the Covid‐19 pandemic, are available at: http://www.teletoile.u‐bordeaux.fr/2020_2021/SympoPACEA_R/index.html#Vis_Accu01_Ap.

## 6 CONCLUSION

In conclusion, the examples given in this paper resonate with other similar experiences as well as findings from neurobiological research to make a strong case for active teaching strategies and “think‐pair‐share methods” that capture student attention and that help to develop their skills and practices (e.g., Owens & Tanner, 2017). Moreover, these experiences encourage use of virtual tools that improve active teaching strategies by extending the potential to immerse students in real‐life professional situations. They reflect the shift to the virtual in evolutionary anthropology courses at its best, namely to access sites, fossils and objects otherwise only virtually available, as well as to release the data hidden within the fossils and objects and preserved on micro‐ or nano‐scopic scales on their surfaces that are otherwise inaccessible. In addition, some theoretical fundamentals are instilled and re‐enforced, such as home‐based self‐learning, which is a prerequisite for the most productive face‐to‐face classes. Used in this way, virtual experiences can help to partially resolve time constraints to free time for face‐to‐face educational opportunities that fieldwork lends, for practical sessions, for mentoring and for seminar classes presenting state of the art advances and future research avenues in the field. In other words, we do not believe that the university should replace in‐person learning with virtual courses but should instead use the substantial opportunities provided by digital options to go beyond traditional coursework and to train students in the societal stakes of the future. Finally, active teaching strategies that support the inclusion of participants can be implemented not only in classroom, laboratory, and fieldwork settings, but also in the conceptualization of learning strategies. Indeed, students are involved in each developmental step offered under the auspices of an immersion program like the ITAP project and thus contribute an active role in the continual improvement of educational practices for future generations.

## CONFLICT OF INTEREST

The authors declare no potential conflict of interest.

## AUTHOR CONTRIBUTIONS

**Priscilla Bayle:** Conceptualization (equal); Data curation (equal); Funding acquisition (equal); Investigation (equal); Methodology (equal); Project administration (lead); Supervision (equal); Visualization (equal); Writing – original draft (lead); Writing – review & editing (equal). **Dominique Armand:** Conceptualization (supporting); Investigation (equal); Methodology (equal); Visualization (supporting); Writing – review & editing (supporting). **Maryelle Bessou:** Conceptualization (supporting); Investigation (equal); Methodology (equal); Visualization (supporting); Writing – review & editing (supporting). **David Cochard:** Conceptualization (supporting); Investigation (equal); Methodology (equal); Visualization (supporting); Writing – review & editing (supporting). **Christine Couture:** Conceptualization (supporting); Investigation (equal); Methodology (equal); Visualization (supporting); Writing – review & editing (supporting). **Marie‐France Deguilloux:** Conceptualization (supporting); Investigation (equal); Methodology (equal); Visualization (supporting); Writing – review & editing (supporting). **Catherine Ferrier:** Conceptualization (supporting); Investigation (equal); Methodology (equal); Visualization (supporting); Writing – review & editing (supporting). **Cathy Haget:** Conceptualization (supporting); Investigation (equal); Methodology (equal); Visualization (supporting); Writing – review & editing (supporting). **Jacques Jaubert:** Conceptualization (supporting); Investigation (equal); Methodology (equal); Visualization (supporting); Writing – review & editing (supporting). **Christopher Knüsel:** Conceptualization (supporting); Investigation (equal); Methodology (equal); Visualization (supporting); Writing – review & editing (supporting). **Stéphanie Martins:** Conceptualization (supporting); Investigation (equal); Methodology (equal); Supervision (supporting); Writing – review & editing (supporting). **Éric Pubert:** Conceptualization (supporting); Investigation (equal); Methodology (equal); Visualization (supporting); Writing – review & editing (supporting). **Stéphane Rottier:** Conceptualization (supporting); Investigation (equal); Methodology (equal); Visualization (supporting); Writing – review & editing (supporting). **Antoine Souron:** Conceptualization (supporting); Investigation (equal); Methodology (equal); Visualization (supporting); Writing – review & editing (supporting). **Cédric Beauval:** Investigation (supporting); Methodology (equal); Visualization (supporting); Writing – review & editing (supporting). **Arnaud Caillo:** Investigation (supporting); Methodology (equal); Visualization (supporting); Writing – review & editing (supporting). **Bruno Dutailly:** Investigation (supporting); Methodology (equal); Visualization (supporting); Writing – review & editing (supporting). **Thomas Girault:** Investigation (supporting); Methodology (equal); Visualization (supporting); Writing – review & editing (supporting). **Malo Hesry:** Investigation (supporting); Methodology (equal); Visualization (supporting); Writing – review & editing (supporting). **François Lacrampe‐Cuyaubère:** Investigation (supporting); Methodology (equal); Visualization (supporting); Writing – review & editing (supporting). **Ronan Ledevin:** Investigation (supporting); Methodology (equal); Visualization (supporting); Writing – review & editing (supporting). **Caroline Masset:** Investigation (supporting); Methodology (equal); Visualization (supporting); Writing – review & editing (supporting). **Miriam Mesa‐Saborido:** Investigation (supporting); Methodology (equal); Visualization (supporting); Writing – review & editing (supporting). **Pascal Mora:** Investigation (supporting); Methodology (equal); Visualization (supporting); Writing – review & editing (supporting). **Xavier Muth:** Investigation (supporting); Methodology (equal); Visualization (supporting); Writing – review & editing (supporting). **Raphaël Pinson:** Investigation (supporting); Methodology (equal); Visualization (supporting); Writing – review & editing (supporting). **Adrien Thibeault:** Investigation (supporting); Methodology (equal); Visualization (supporting); Writing – review & editing (supporting). **Marc Thomas:** Investigation (supporting); Methodology (equal); Visualization (supporting); Writing – review & editing (supporting). **Nicolas Vanderesse:** Investigation (supporting); Methodology (equal); Visualization (supporting); Writing – review & editing (supporting). **Jean‐Guillaume Bordes:** Conceptualization (equal); Data curation (equal); Funding acquisition (equal); Investigation (equal); Methodology (equal); Project administration (supporting); Supervision (equal); Visualization (equal); Writing – original draft (supporting); Writing – review & editing (equal).

## Data Availability

Educational videos and 3D models are available directly from the above‐mentioned websites. Online exercises using these multimedia sources are available to Master's students on the educational platform of the University of Bordeaux and are more widely available upon request to the corresponding authors. The “Virtual Sirogne” application will be available online after publication of the results of the ongoing site excavation.
